# Impact of Age and Years in the Fire Service on Firefighter Health and Physical Performance Outcomes †

**DOI:** 10.3390/healthcare13161946

**Published:** 2025-08-08

**Authors:** Jisun Chun, Michael J. Conner, Jacob A. Mota, Brian Newman, J. Jay Dawes, Steven E. Martin, Drew Edward Gonzalez

**Affiliations:** 1 Tactical Athlete Research Unit, Department of Kinesiology and Sports Management, Texas A&M University, College Station, TX 77843, USA; chunjs3112@tamu.edu (J.C.); semartin@tamu.edu (S.E.M.); 2 Front Line Mobile Health, PLLC, Granbury, TX 76048, USA; mike@frontlinemobilehealth.com; 3 Neuromuscular and Occupational Performance Laboratory, Department of Kinesiology and Sport Management, Texas Tech University, Lubbock, TX 79409, USA; jacob.mota@ttu.edu; 4 Fort Worth Fire Department, Fort Worth, TX 76115, USA; brian.newman@fortworthtexas.gov; 5 School of Kinesiology, Applied Health, and Recreation, Oklahoma State University, Stillwater, OK 74078, USA; jay.dawes@okstate.edu; 6 Tactical Fitness and Nutrition Lab, Oklahoma State University, Stillwater, OK 74078, USA; 7 Department of Kinesiology & Sport Management, Sydney and JL Huffines Institute for Sports Medicine and Human Performance, Texas A&M University, College Station, TX 77843, USA; 8 Occupational, Performance, and Nutrition Laboratory, Department of Kinesiology, Sam Houston State University, Huntsville, TX 77341, USA

**Keywords:** cardiometabolic health, heart disease, blood lipids, tactical athlete, firefighting

## Abstract

**Background**: Age is considered an independent cardiovascular disease (CVD) risk factor, but limited data exist concerning the impact of age and years in the fire service on firefighter health and physical performance. **Objectives**: We assessed the impact of age and years in the fire service on structural firefighter health and physical performance. **Methods:** 142 firefighters completed an annual clinical assessment. General linear model (GLM) multivariate and univariate analyses were performed across age groups, with years of fire service experience as a covariate. Partial Eta squared (η_p_^2^) values for the GLM statics and Cohen’s d for pairwise comparisons were used to assess effect size. **Results**: GLM analyses revealed statistically significant differences (*p* < 0.05) for several demographic, body composition, blood biomarker, and physical performance parameters. Pairwise comparisons revealed that younger firefighters displayed more favorable health and fitness profiles than their older counterparts. Interestingly, when accounting for years of fire service experience, differences for only the following variables remained: body mass index, waist circumference, lean mass, visceral adipose tissue, triglycerides, cardiopulmonary exercise test time-to-exhaustion, and VO_2_max. **Conclusions**: These findings suggest differences in CVD risk biomarkers, physical fitness levels, and body composition metrics across age groups. However, years of fire service experience appears to have a greater impact on these variables, suggesting that firefighters’ time in the fire service may accelerate the effects of aging on these outcomes. While age-specific health interventions are recommended to manage and mitigate CVD risk effectively, there also should be consideration for the years of fire service.

## 1. Introduction

Structural firefighting is a physically demanding and high-stress occupation associated with an elevated risk of cardiovascular disease (CVD), among other diseases and disorders (i.e., cancer and depression) [1,2,3]. Notably, CVD, particularly sudden cardiac death, is the primary cause of premature mortality, responsible for nearly 45–50% of all firefighter fatalities recorded over the past four decades [2,4,5,6]. In addition, data from the United States Fire Administration report on firefighter fatalities suggests ≈97% of the line-of-duty deaths over a 30-year period occurred in firefighters >30 years of age [7]. The activities inherent in firefighting, including roof ventilation, dry and charged hose deployment, and rescue operations (e.g., victim search and rescue), impose acute physiological stress [8,9], likely accelerating the process of aging and worsening existing comorbidities associated with CVD [10].

Firefighters with lower cardiorespiratory and musculoskeletal fitness and poor body composition (i.e., greater body fat/fat mass with lower lean mass) exhibit worse CVD risk factors than their more fit/healthier counterparts [11]. For instance, McAllister and colleagues [12] found that although the more fit firefighters expressed a healthier body mass index (BMI; kg/m^2^), lower blood lipid concentrations, and a 46% higher maximal oxygen uptake (VO_2_max), there was no difference in age (*p* = 0.52; low-fit group, n = 27, 38 ± 9 years; high-fit group, n = 19, 36 ± 9 years). However, due to a small sample size, the impact of age on the outcome variables may have been masked [12]. Mathias and colleagues [13] assessed changes in CVD risk factors among 656 firefighters over five years. Following the five-year period, 50% of the sample gained weight (6.6 ± 0.2 kg, *p* < 0.001) and exhibited increased total cholesterol (TC; 12.9 ± 1.8 mg/dL, *p* < 0.001), low-density lipoprotein cholesterol (LDL-C; 11.1 ± 1.6 mg/dL, *p* < 0.001), and blood glucose levels 2.9 ± 0.7 mg/dL, *p* < 0.001), accompanied by lower high-density lipoprotein (HDL-C; 1.3 ± 0.4 mg/dL, *p* < 0.01) concentrations. Furthermore, the participants had an increase in their 10-year risk of having a CVD event (2.6 ± 0.2%, *p* < 0.001) based on data from the Framingham Heart and the Framingham Offspring study [14]. Considering these findings, as one ages, the risk for CVD increases, and this is likely influenced by the years spent in the occupation, which could also impact this risk. In fact, early work by Saupe and colleagues [15] found that firefighters displayed poorer health and fitness profiles (i.e., lower maximal aerobic capacity) between age groups 40 to 45, 50 to 55, and 60 to 65, supporting this notion.

Although it is well recognized that CVD risk rises with age in the general population [16,17,18], there is a paucity of data regarding how age-related changes affect the health and fitness profiles of firefighters. To date, several studies have assessed the impact of age on a firefighter’s physical abilities related to the occupation [19,20,21,22,23]. For instance, Parpa and Michaelides [20] assessed age-related differences among firefighters performing six firefighting tasks, with three age groups: 20 to 30, 31 to 40, and 41 to 50 years of age. They found that age had a statistically significant effect on body fat percentage, waist circumference, and waist-to-hip ratio, in addition to the overall time spent completing individual and a battery of firefighting tasks [20]. Furthermore, Saari et al. [21] found that older firefighters (over 37 years old) displayed lower physical capabilities, as evidenced by the fact that it took them 8.8% longer to complete a firefighting simulation assessment compared to younger firefighters (under 37 years old). On the contrary, Bennet and colleagues [19] suggested that other factors, such as self-reported physical activity levels and whether one is classified as overweight or obese, had more influence on maintaining cardiorespiratory fitness levels than age.

Compared to the general public, age-matched firefighters exhibit a higher risk of acute myocardial infarction across various age groups (i.e., 20 to 29, 30 to 39, etc.), which may suggest that the stressors of firefighting accelerate the aging process and increase the risk of CVD-related events [24]. While considerable work has examined the impact of age on firefighting abilities [19,20,21,22,23], along with a handful of studies on health and physical fitness-related outcomes [12,13,15,24], to our knowledge, no study has assessed the impact of age while considering years of fire service experience. It is plausible that the longer a firefighter is in the fire service, the greater their CVD risk. Therefore, we retrospectively analyzed data collected from an annual clinical assessment (in 2024) among firefighters to evaluate the impact of age and years of fire service experience on health (e.g., body composition and blood biomarkers) and physical performance (e.g., push-ups and sit-ups) of firefighters.

## 2. Materials and Methods

### 2.1. Participants and Experimental Design

**Figure 1:**
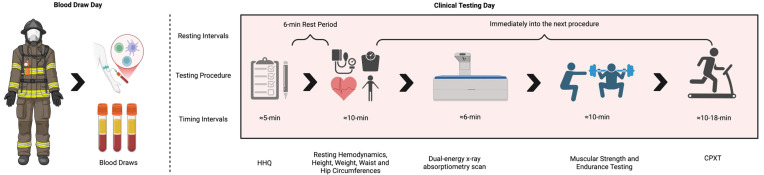
Annual clinical testing battery. HHQ = Health History Questionnaire; CPXT = Cardiopulmonary Exercise Test.

Retrospective data for one-hundred and forty-two (n = 142; age = 33.7 ± 8.9 years; weight = 95.1 ± 12.8 kg; height = 179.4 ± 9.6 cm; BMI = 29.7 ± 6.7 kg/m^2^) career structural firefighters from a single fire department in the South-Central region of the United States were analyzed. Consistent with the Strengthening the Reporting of Observational Studies in Epidemiology (STROBE) guidelines for transparent reporting of participant characteristics [25], we included all participants who met the inclusion criteria, including one female firefighter—the only female in the participating fire department. As sex was not a variable of interest in the present analysis and subgroup comparisons were not feasible, the results primarily reflect those of male firefighters. However, the inclusion of this participant is unlikely to have impacted group-level statistics, and we chose to retain her data to maximize transparency and completeness. Written informed consent was obtained before the participants completed a series of general health, wellness, and lifestyle history questionnaires, which screened for signs, symptoms, and diagnosis of cardiometabolic and blood diseases/disorders. Inclusion criteria included (1) being between the ages of 18 and 65 at the time of consent, (2) being a current career firefighter of the fire department, and (3) providing informed consent. Exclusion criteria included individuals outside the specified age range, those not affiliated with the fire department, and individuals currently receiving pharmacotherapy interventions for hypercholesterolemia/dyslipidemia, hypertension, or other cardiometabolic-related conditions (e.g., statins, metformin, beta-blockers). Data were collected by the Sydney and J.L. Huffines Institute for Sports Medicine and Human Performance Physical Readiness & Testing Facility Team, under the supervision of Dr. Steven Martin, during the fire department’s annual clinical testing assessment in the spring and summer of 2024. Additionally, these annual clinical assessments are conducted under the supervision of a medical doctor. The clinical testing took place over two days, during which the firefighters had their blood drawn on one day during their shift rotation (i.e., rotating on and off shift) and then completed a battery of clinical exercise physiology testing on the second day of testing (Figure 1). All testing and study procedures were conducted in full compliance with the Declaration of Helsinki and were approved by the Institutional Review Board of Texas A&M University (IRB2023-0957D).

### 2.2. Rationale for Age Groups

Participants were grouped into the following age categories for analysis: 20–29 years, 30–39 years, and 40 years and older. These categories were chosen to reflect meaningful decade-based differences in physiological aging while also balancing the sample size across groups. The ≥40 group included all participants aged 40 and above, consisting of seven participants aged 50–59 and one participant aged 60 and older, to maintain statistical power and prevent underpowered subgroup analyses due to small sample sizes in the older age brackets. Participants aged 18–19 were not included in the sample and, therefore, are not represented in the analysis.

### 2.3. Testing Overview and Procedures

The procedures subsequently described have been previously detailed [11]. The annual clinical testing took place over two days, including a bio-sample collection and laboratory testing days. During the bio-sample collection day, the firefighters reported to a predetermined blood draw site at one of the centrally located fire stations at 7:20 in the morning and donated a fasted (≥12 h) blood sample. Then, on a separate day, the firefighters reported to an on-campus testing facility and underwent a battery of clinical testing, including (1) assessments of resting hemodynamics, body composition, and anthropometrics, (2) muscular strength, endurance, and flexibility testing, and (3) a maximal cardiopulmonary exercise test (CPXT), where VO_2_max was estimated from the Foster equation using time-to-exhaustion (TTE) [26].

#### 2.3.1. Demographics, Anthropometrics, and Body Composition Assessments

Participants completed a series of health, wellness, and lifestyle questionnaires, wherein age was recorded. Following this, height and body mass were assessed using a mechanical eye-level physician’s scale and height rod. Next, a laboratory research technician took waist and hip circumference measures following World Health Organization procedures [27,28]. Finally, the participants underwent a dual-energy x-ray absorptiometry scan (DEXA; Hologic Horizon A, Marlborough, MA) to assess their fat mass, fat-free mass, body fat percentage, visceral adipose tissue, and android and gynoid body fat distribution. Lastly, we calculated the Framingham Risk Score for Hard Coronary Heart Disease (i.e., 10-year risk of having a CVD event) using demographic data (i.e., age, sex), health history screening questionnaire responses (i.e., smoker history, blood pressure medication), and clinical data (i.e., blood lipids [TC and HDL-C] and systolic blood pressure values) to estimate the 10-year risk of a first CVD event among ≥30-year-olds [13,14].

#### 2.3.2. Physical Performance Parameters

Following the collection of demographic, anthropometric, and body composition assessment data, participants completed a sit-and-reach assessment using standard American College of Sports Medicine (ACSM) procedures [29]. Then, the participants performed an assessment involving 1 min of sit-ups and push-ups, wherein they had to complete as many repetitions as possible with proper form [29]. Finally, the participants’ cardiorespiratory fitness was assessed during a symptom-limited CPXT using the Bruce protocol. VO_2_max was estimated from the Foster equation [26]. The CPXT was completed on a standard TM65 treadmill with a 12-lead electrocardiogram system (Quinton Q Stress System, Cardiac Science Corporation, Bothell, WA, USA).

#### 2.3.3. Blood Collection and Analysis Procedures

Before the bio-sample collection day, participants were instructed to fast for at least 12 h. Following standard phlebotomy procedures, ≈8.5 mL of fasted blood was obtained into 2 × 8.5 mL serum separation tubes (SSTs) and 1 × 4 mL K2 Ethylenediaminetetraacetic Acid (EDTA) tube (Becton, Dickinson and Company, Franklin Lakes, New Jersey, USA) from the antecubital fossa by certified phlebotomists [28]. Immediately after collection, the bio-samples were gently rotated to mix the contents of the tubes, allowed to rest and clot at room temperature for ≈30 min, and then transported to a Biosafety Level 2 (BSL-2) laboratory and a commercial laboratory (Clinical Pathology Labs Inc., Austin, TX, USA) within 1 h of collection. Two SSTs and one EDTA tube were transported to the commercial laboratory for assessment of TC, triglycerides (TAG), HDL-C, LDL-C, fasting plasma glucose, fasting plasma insulin, apolipoprotein B (ApoB), and hemoglobin-A1c (HbA1c). Both fasting plasma glucose and insulin were used to calculate the Homeostatic Model Assessment for Insulin Resistance (HOMA-IR) using the following formula, with units in the International System of Units: HOMA-IR = (fasting glucose concentration [mmol/L] × fasting insulin concentration [pmol/L])/405.

### 2.4. Statistical Analysis

All statistical procedures were performed with the IBM^Ⓡ^ Version 29 SPSS^Ⓡ^ statistical analysis software (IBM Corp., Armonk, NY, USA). A general linear model (GLM) for multivariate and univariate analyses was used to assess differences across age groups (i.e., 20 to 29, 30 to 39, and 40+), with and without years of fire service experience as a covariate, for demographic, body composition, blood biomarkers, and fitness parameters. The type I error (*p*-level) probability was set a 0.05 or less. Data are presented as means ± standard deviations (SD) or means ± standard error (SE) when accounting for years of fire service experience as a covariate. Partial Eta squared (η_p_^2^) values were used to assess effect size, where values of 0.01 (small effect), 0.06 (medium effect), and 0.14 (large effect) for the GLM statics, while pairwise comparisons between age groups effect sizes were calculated using Cohen’s d and interpreted as follows: 0.2 = small, 0.5 = medium, 0.8 = large, 1.2 = very large, 2.0 = huge. To assess potential multicollinearity between age and years of fire service experience, variance inflation factors (VIFs) were calculated using linear regression models that mirrored the GLM predictor structure. The VIFs were interpreted as <2 = low multicollinearity, 2 to 5 = moderate multicollinearity, and >5 = high multicollinearity. Results from the GLM multivariate and univariate analyses, including covariate-adjusted univariate outcomes, are presented in the tables. The figures display the pairwise comparison outcomes for parameters that showed a statistically significant effect of age, along with the covariate-adjusted results in the univariate analyses.

## 3. Results

### 3.1. Demographics Data

**Figure 2:**
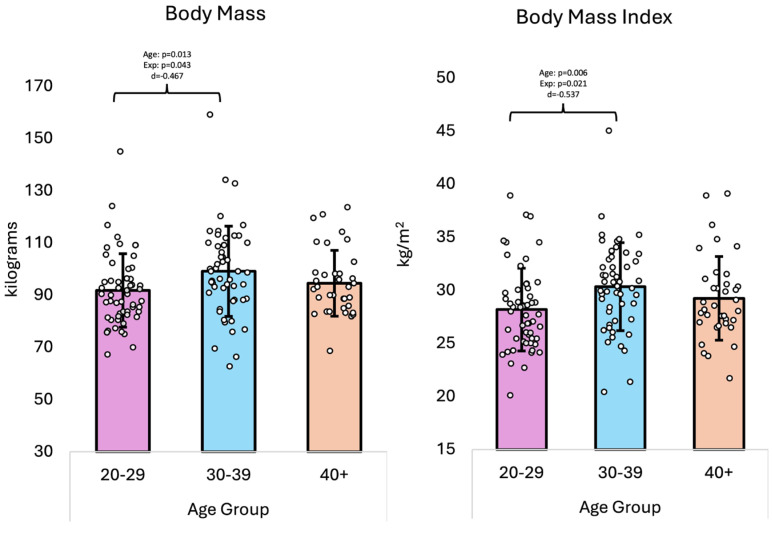
Body mass and BMI data (means ± standard deviations); ‘Age’ denotes the effect of age; ‘Exp’ denotes the effect of age when adjusting for years of fire service experience as a covariate; ‘d’ denotes Cohen’s d effect size; kg = kilograms; m^2^ = meters squared.

The overall GLM multivariate analysis (Table 1) revealed no significant differences for the age group (*p* = 0.060, η^2^ = 0.043). Univariate analysis showed differences in body mass (*p* = 0.043, η^2^ = 0.044) and BMI (*p* = 0.023, η^2^ = 0.053). Pairwise comparisons found that the 20–29 group differed from the 30–39 group in that they had lower body mass (*p* = 0.013, d = −0.467) and BMI (*p* = 0.006, d = −0.537) values. However, there were no differences noted between the 20–29 and 40+ group, nor the 30–39 and 40+ group (*p* > 0.05). When accounting for years of fire service experience as a covariate, the overall GLM multivariate analysis demonstrated no differences in demographics across age groups. Univariate analysis showed differences only in BMI. The pairwise comparisons revealed that the 20–29 group differed from the 30–39 group in that they had lower BMI values (*p* = 0.021). Figure 2 shows the body mass and BMI data. Regarding the Framingham CVD risk score, a separate GLM univariate analysis was conducted, revealing an effect for age (*p* < 0.001, η^2^ = 0.223) for the 10-year risk of having a CVD event. Notably, there was a 3.07 percentage point greater (or greater than fourfold or ≈402%) in the 10-year risk for the 40+ age group compared to the 30–39 group. However, when adjusting for years of experience as a covariate, a non-statistically significant effect of age (*p* = 0.104, η^2^ = 0.032) was found with a statistically significant effect of years of fire service experience (*p* = 0.003, η^2^ = 0.107). It is important to note that this 10-year risk of having a CVD event could not be calculated for those in the 20–29 age group as their age group is not considered within the Framingham Score calculation.

### 3.2. Anthropometrics and Body Composition

The overall GLM multivariate analysis (Table 2) indicated differences in the anthropometrics and body composition parameters across age groups (*p* < 0.001, η^2^ = 0.201). Univariate analysis revealed differences in waist circumference (*p* < 0.001, η^2^ = 0.118), waist-to-hip ratio (*p* < 0.001, η^2^ = 0.151), body fat percentage (*p* = 0.003, η^2^ = 0.078), fat mass (*p* = 0.020, η^2^ = 0.055), lean mass (*p* = 0.021, η^2^ = 0.054), android body fat distribution (*p* < 0.001, η^2^ = 0.122), visceral adipose tissue (*p* < 0.001, η^2^ = 0.226), but not for hip circumference (*p* = 0.217, η^2^ = 0.022) or gynoid body fat distribution (*p* = 0.055, η^2^ = 0.041). Pairwise comparisons showed that the 20–29 group differed from the 30–39 and 40+ groups in that they had lower measures of waist circumference, waist-to-hip ratios, body fat percentage, fat mass, android body fat distribution, and visceral adipose tissue. The pairwise comparisons also found that the 30–39 group differed from the 40+ group, as the 30–39 group displayed greater lean mass and lower visceral adipose tissue than the 40+ group.

**Figure 3:**
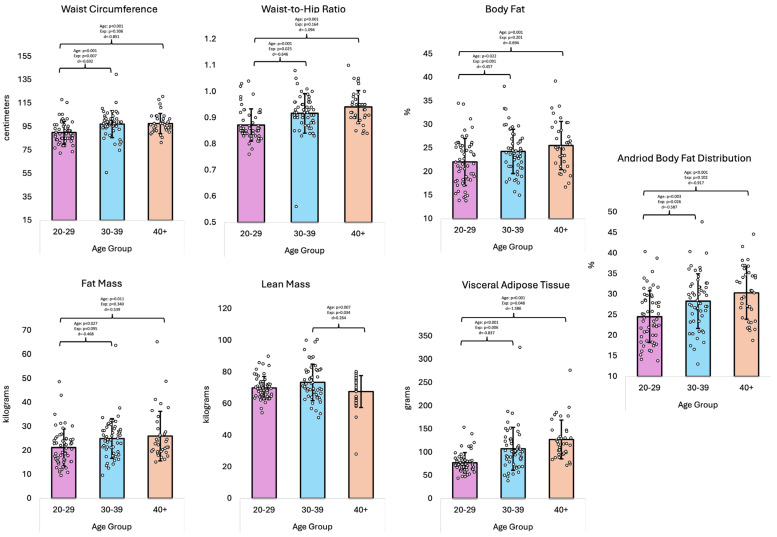
Body composition data (means ± standard deviations); ‘Age’ denotes the effect of age; ‘Exp’ denotes the effect of age when adjusting for years of fire service experience as a covariate; ‘d’ denotes Cohen’s d effect size; % = percentage.

When accounting for years of fire service experience as a covariate, the overall GLM multivariate analysis indicated no differences in anthropometrics and body composition across age groups (*p* = 0.231, η^2^ = 0.079). Univariate analysis revealed differences in waist circumference (*p* = 0.020, η^2^ = 0.055), lean mass (*p* = 0.032, η^2^ = 0.049), and visceral adipose tissue (*p* = 0.021, η^2^ = 0.054). The pairwise comparisons found that the 20–29 group differed from the 30–39 group in that they had lower waist circumferences and visceral adipose tissue. The pairwise comparisons also found that the 30–39 group differed from the 40+ group, as they displayed higher lean mass. Figure 3 shows the body composition data.

### 3.3. Blood Biomarkers

Regarding the blood biomarkers (Table 3), the overall GLM multivariate analysis revealed significant differences across age groups (*p* = 0.001, η^2^ = 0.148). Univariate analysis revealed that there were differences for TC (*p* = 0.001, η^2^ = 0.099), LDL-C (*p* < 0.001, η^2^ = 0.106), ApoB (*p* < 0.001, η^2^ = 0.158), TAG (*p* < 0.001, η^2^ = 0.111), insulin (*p* = 0.047, η^2^ = 0.044), HbA1c (*p* = 0.026, η^2^ = 0.052), and HOMA-IR (*p* = 0.024, η^2^ = 0.053), while no differences were noted for HDL-C (*p* = 1.114, η^2^ = 0.031) or blood glucose (*p* = 0.138, η^2^ = 0.029). Pairwise comparisons found that the 20–29 differed from the 30–39 and 40+ groups in that they had lower concentrations of TC, LDL-c, ApoB, and TAG, while the 20–29 group differed only from the 40+ group with lower insulin and blood glucose concentrations, in addition to lower HOMA-IR and HbA1c levels. Pairwise comparisons also found that the 30–39 group differed from the 40+ group, wherein the 30–39 group displayed lower concentrations of ApoB and HbA1c levels.

**Figure 4:**
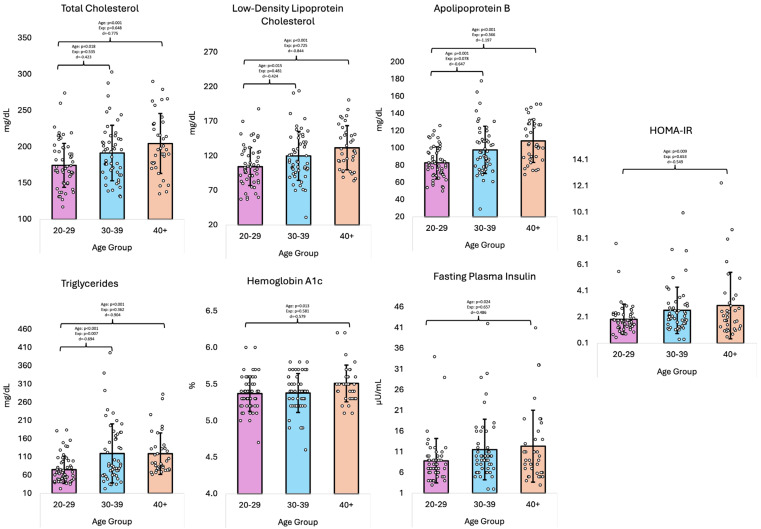
Blood biomarker data (means ± standard deviations); ‘Age’ denotes the effect of age; ‘Exp’ denotes the effect of age when adjusting for years of fire service experience as a covariate; ‘d’ denotes Cohen’s d effect size; dL = deciliter; HOMA-IR = Homeostatic Model Assessment for Insulin Resistance; mg = milligrams; mL = milliliters; µU = microunits; % = percentage.

When accounting for years of fire service experience as a covariate, the overall GLM multivariate analysis revealed no significant differences in blood biomarkers across age groups (*p* = 0.132, η^2^ = 0.090). Univariate analysis showed differences for only TAG (*p* = 0.019, η^2^ = 0.057), with pairwise comparisons indicating that the 20–29 group had lower concentrations of TAG compared to the 30–39 group (*p* = 0.007). Figure 4 shows the blood biomarker data.

### 3.4. Physical Performance Parameters

The overall GLM multivariate analysis indicated significant differences in fitness parameters across age groups (*p* < 0.001, η^2^ = 0.180). Univariate analysis revealed that there were differences for CPXT TTE (*p* < 0.001, η^2^ = 0.206), VO_2_max (*p* < 0.001, η^2^ = 0.211), push-ups (*p* < 0.001, η^2^ = 0.160), and sit-and-reach (*p* < 0.001, η^2^ = 0.117), while no differences were noted for sit-ups (*p* = 0.085, η^2^ = 0.036) and handgrip strength (*p* = 0.116, η^2^ = 0.031). Pairwise comparisons found that the 20–29 group differed from the 30–39 and 40+ groups in that they had higher CPXT TTE and VO_2_max values, in addition to greater flexibility (as measured by the sit-and-reach test) and the ability to perform more repetitions on the push-up test. The 20–29 group also differed from the 30–39 group in their ability to perform more repetitions on the sit-up test, with no difference noted between the 20–29 group and the 40+ group (20–29 group: 44.4 ± 7.9; 40+ group: 41.1 ± 9.6; p = 0.117). Furthermore, the pairwise comparisons found no difference in fitness parameters between the 30–39 and 40+ groups (*p* > 0.05).

**Figure 5:**
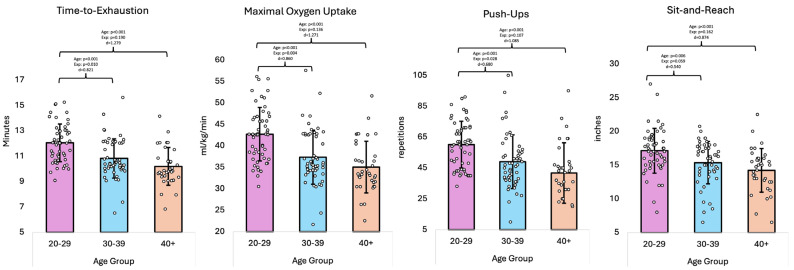
Physical performance data (means ± standard deviations); ‘Age’ denotes the effect of age; ‘Exp’ denotes the effect of age when adjusting for years of fire service experience as a covariate; ‘d’ denotes Cohen’s d effect size; kg = kilograms; ml = milliliters; µU = microunits.

When accounting for years of fire service experience as a covariate, the overall GLM multivariate analysis revealed differences in fitness parameters across age groups (*p* = 0.008, η^2^ = 0.096). Univariate analysis showed that there were differences for CPXT TTE (*p* = 0.034, η^2^ = 0.049) and VO_2_max (*p* = 0.016, η^2^ = 0.060), but no differences were found for push-ups (*p* = 0.082, η^2^ = 0.036), sit-ups (*p* = 0.062, η^2^ = 0.040), sit-and-reach (*p* = 0.158, η^2^ = 0.027), or handgrip strength (*p* = 0.116, η^2^ = 0.031). Pairwise comparisons revealed that the 20–29 group differed significantly from the 30–39 group for both CPXT TTE (*p* = 0.010) and VO_2_max (*p* = 0.004), with no other differences between groups noted. Table 4 displayed the fitness parameter data when accounting for years of fire service experience as a covariate. Figure 5 shows the fitness parameter data.

### 3.5. Multicollinearity Assessment

The multicollinearity assessment revealed consistent VIFs for the age and years of experience across models (VIF ≈ 3.739 to 3.931), which suggests moderate but acceptable shared variance for these independent variables. As such, where changes in predictor significance were noted across the demographic, body composition, blood biomarkers, and physical performance parameters, it was unlikely to be solely attributable to multicollinearity.

## 4. Discussion

The present study aimed to assess the impact of age and years of fire service experience on the health and physical performance of career firefighters. The study’s main findings revealed statistically significant differences in several demographics, body composition, blood biomarkers, and physical performance parameters with the 20–29-year-old firefighters displaying more favorable health and physical fitness profiles than the 30–39 and 40+ age groups. While aging is well known to contribute to declines in health and physical performance, our findings suggest that years of fire service experience partially overlap with the age-related effects and may serve as a proxy for cumulative occupational burden. Specifically, when adjusting for years of experience, many of the initially observed age-related differences in health and performance outcomes were no longer statistically significant. This indicates that the variance shared between age and service time may obscure which factor is most influential. However, certain variables (e.g., VO_2_max, CPXT time-to-exhaustion, visceral adipose tissue) remained statistically significantly affected even after covariate adjustment, indicating a potential dual influence of both aging and occupational exposure—especially when considering the moderate VIFs (3.93). Notably, years of service did not consistently predict worse health outcomes on its own, but in some cases (e.g., ApoB, LDL-C, TC), one’s years of fire service experience did show stronger associations than age, underscoring its possible contribution to cumulative risk. These findings support a model in which the cumulative stress of firefighting may exacerbate the natural effects of aging, particularly concerning cardiorespiratory fitness and body composition parameters. They also emphasize the necessity for tailored strategies that address both age and service-related stress.

Aging populations are susceptible to CVD, among other cardiometabolic/chronic diseases, and this risk may be further exacerbated by factors, such as obesity and poor physical fitness levels [17,30]. In the case of a firefighter, the natural aging process likely is accelerated due to the increase and repeated exposure to hazardous stressors that induce physiological stress (i.e., elevations in cortisol), oxidative stress, and inflammation [31]. For instance, Jeung and colleagues [24] found that acute myocardial infarctions were more common among younger firefighters than older firefighters (e.g., hazard risk for 20–29 = 2.267 vs. 40+ firefighters = 1.510 to 1.530), suggesting that the cumulative occupational burden may led to a greater susceptibility of a CVD event even at a younger age. The authors speculated on possible explanations for the higher hazard risk of acute myocardial infarction for the younger firefighter, suggesting either post-traumatic stress disorder, closer proximity to traumatic events, the “healthy worker survival effect” (i.e., older firefighter removing themselves from traumatic exposure), or perception medications may play a role. Considering these explanations, it seems plausible that the younger firefighters are faced with more strenuous occupational exposures and, while likely more fit than their older counterparts, face a compounding effect of numerous stress exposure that leave them susceptible to a CVD event. However, it is important to note that Jeung and colleagues [24] did not directly assess for years of fire service experience. Recently, Martin and colleagues [32] assessed the impact of years of service as a mediator on physical fitness outcomes among 1281 firefighters and found that years of service exacerbated the decline in musculoskeletal strength (primarily core strength on the curl-up test) and endurance (assessed by the firefighter’s ability to perform pull-ups and push-ups), independent of age. Our findings appear to align with those of Jeung and colleagues [24] and Martin and colleagues [32] in that years of fire service experience likely have a unique effect on several health and fitness outcomes, suggesting that the longer one is in the fire service, the greater their risk may be for CVD. Therefore, even if the firefighter is young and fit, their risk of a CVD event may be influenced by the cumulative time within the fire service. However, the novelty of our study lies in our direct assessment of the impact of years of experience on health and fitness-related outcome variables. Overall, these patterns indicate that years of service may not only account for some of the variance attributed to aging but may also represent a unique health risk driven by occupation, independent of chronological age.

Age does play a role in CVD risk [33], and our findings suggest that age also influences several health and physical performance parameters, including BMI, waist circumference, lean mass, visceral adipose tissue, triglycerides, CPXT TTE exercise test time-to-exhaustion, and VO_2_max. The younger firefighters displayed lower BMI, waist circumference, visceral adipose tissue, and triglyceride levels while having greater lean mass and higher CPXT TTE and VO_2_max values than their older counterparts, even after adjusting for years of fire service experience. This finding aligns with previous reports that demonstrate an age-related decline in parameters, such as cardiorespiratory fitness. For example, Baur et al. [34] found that cardiorespiratory fitness level declined with age among 804 career, male firefighters. Not surprisingly, this decline was mitigated among those with greater lean mass [34]. Another study by Bode et al. [35] found that among 4453 firefighters, those who were older displayed higher triglycerides, in addition to higher concentrations of TC and blood glucose. The authors concluded that with each decade of life, firefighters will likely display increases in BMI, which coincides with high blood pressure and triglycerides, and low HDL-C [35]. Considering these findings by Baur et al. [34] and Bode et al. [35] in relation to ours, it is clear that age has an effect on a firefighter’s health and physical fitness/abilities, and tailored “anti-aging” strategies are warranted to combat the deleterious effects of the aging process. In some cases, years of fire service experience does impact the health and fitness parameters more than one’s age, but with respect to others, age appears to have a unique effect. Taken together, firefighters should aim to increase and/or maintain higher levels of lean skeletal muscle mass and cardiorespiratory fitness while monitoring other markers of CVD risk (i.e., through routine clinical assessments/physicals) to ensure they are at a lower risk of a CVD event, especially as they age or serve more time as a firefighter. These distinctions emphasize that neither age nor experience alone fully explains the trajectories of health risks. Instead, certain outcomes are likely driven by biological aging, others by occupational exposure, and some by both. Given the combined impact of physiological aging and service-related stressors, proactive strategies are necessary to mitigate risk—especially for parameters that are influenced by both factors, such as cardiorespiratory fitness and body composition. Furthermore, healthcare professionals working with firefighters should promote tailored interventions (i.e., dietary/nutritional interventions, such as the Mediterranean diet or supplemental creatine) to combat the natural aging process, as well as advocate for maintaining healthy levels of fitness and body composition profiles.

The present study has limitations. First, our dataset is from a larger longitudinal study and the data are of a convenience sample, meaning that this sample is non-probabilistic, and strict interpretations may not be appropriate. Therefore, the statistical tests should be interpreted and understood relative to this study’s sample. Second, we did not collect overtime hours, which may have an influence on the cumulative occupational burden faced in the fire service (i.e., working more overtime hours may lead to a greater occupational exposure overtime). Future studies should consider tracking overtime hours, as well as secondary job hours (hours per week), to gain a better understanding of the cumulative effect of the occupational burden placed on the firefighter. Third, the annual clinical testing for which this dataset is from is not mandatory testing; therefore, there may be a “healthy worker effect”, wherein those who are more fit and healthier may have a greater desire to partake in the annual clinical testing [36,37]. This could potentially mean those who are at even higher risk of CVD were missed during data collection. Lastly, our sample only had one female firefighter included due to a limited number within the fire department. Therefore, our results primarily reflect men.

## 5. Conclusions

Our findings highlight that both chronological age and years of fire service experience influence firefighters’ cardiometabolic health, physical performance, and cardiovascular disease CVD) risk. While age-related declines are well documented, our data suggest that cumulative occupational exposure may worsen or, in some cases, mimic these effects. Specifically, younger firefighters with more years of service may show risk profiles akin to those of their older counterparts, highlighting the combined effects of biological aging and occupational stress. Not all outcomes were influenced equally. In some cases, age was the stronger predictor, while in others, years of experience showed a greater association. This complexity reinforces the need for targeted, individualized preventive strategies that preserve or enhance lean muscle mass, cardiorespiratory fitness, and metabolic health. Routine clinical assessments and continuous monitoring of fitness and performance parameters are warranted to reduce long-term risk and promote occupational readiness throughout a firefighter’s lifespan.

## Figures and Tables

**Table 1:** Demographic Results.

Variable	Age Group	*n*	Effect of Age				Effect of Age with Years of Experience Covariate ^a^			
			Mean	Mean	Univariate Results		Mean	Source	Univariate Results	
			(SD)	(SE)	*p*-Value	η_p_^2^	(SD)		*p*-Value	η_p_^2^
Body Mass	20–29 years	54	91.8 ± 14.0	91.8 ± 2.0	0.043	0.044	92.6 ± 2.6	Exp	0.635	0.002
(kg)	30–39 year	52	99.2 ± 17.3	99.2 ± 2.1			99.3 ± 2.1			
	40+	36	94.6 ± 12.6	94.6 ± 2.5			93.3 ± 3.7	Age	0.053	0.042
	Total	142	95.2 ± 15.2	95.2 ± 1.3			95.1 ± 1.3			
Height	20–29 years	54	180.5 ± 6.2	180.5 ± 0.9	0.905	0.001	180.7 ± 1.1	Exp	0.697	0.001
(cm)	30–39 year	52	180.4 ± 7.5	180.4 ± 0.9			180.5 ± 0.9			
	40+	36	179.9 ± 5.3	179.9 ± 1.1			179.4 ± 1.6	Age	0.844	0.002
	Total	142	180.3 ± 6.4	180.3 ± 0.6			180.2 ± 0.6			
Body Mass Index	20–29 years	54	28.2 ± 3.9	28.2 ± 0.5	0.023	0.053	28.3 ± 0.7	Exp	0.755	0.001
kg/m^2^	30–39 year	52	30.3 ± 4.1	30.3 ± 0.6			30.4 ± 0.6			
	40+	36	29.3 ± 3.9	29.3 ± 0.7			29.0 ± 1.0	Age	0.037	0.047
	Total	142	29.3 ± 4.1	29.3 ± 0.3			29.2 ± 0.4			

Data are expressed as means ± standard deviations (SD) with grand mean results shown as means ± standard error (SE) for the the age group effect and the effect when considering years of fire service experience as a covariate. Age = effect of age; Exp = effect of years of fire service experience; η_p_^2^ = partial eta squared effect size; n = sample size; SD = standard deviation; SE = standard error; kg = kilograms; cm = centimeters; m^2^ = meters squared. 20–29 years is the age group of those 20 to 29 years old; 30–39 years is the age group of those 30 to 39 years old; 40+ is the age group of those 40 years old or older, including those between 50–59 (7 participants) and 60+ (1 participant). Effect of Age: General linear model analysis revealed no significant overall Wilk’s Lambda for age group (*p* = 0.060, η_p_^2^ = 0.043). When considering years of fire service as a covariate, the general linear model analysis revealed no significant overall Wilk’s Lambda for age group (*p* = 0.086, η_p_^2^ = 0.040). Univariate p-levels are listed for between-subject effects with η_p_^2^ effect size values of 0.01–0.05 = small, 0.06–0.13 = medium, and >0.14 = large. Significance was determined via pairwise comparison, with LSD posthoc adjustment, and Cohen’s d effect size. ^a^ Covariates appearing in the model are evaluated at the following values: Years of fire service experience = 8.7465. Note: these results include data from one female firefighter.

**Table 2:** Body Composition Results.

Variable	Age Group	*n*	Effect of Age				Effect of Age with Years of Experience Covariate ^a^			
			Mean	Mean	Univariate Results		Mean	Source	Univariate Results	
			(SD)	(SE)	*p*-Value	η_p_^2^	(SD)		*p*-Value	η_p_^2^
Hip Circumference	20–29 years	54	103.3 ± 9.2	103.3 ± 1.2	0.217	0.022	103.4 ± 1.5	Exp	0.914	0.000
(cm)	30–39 year	52	106.2 ± 8.8	106.2 ± 1.2			106.2 ± 1.2			
	40+	36	103.9 ± 7.8	103.9 ± 1.5			103.8 ± 2.2	Age	0.224	0.021
	Total	142	104.5 ± 8.8	104.5 ± 0.7			104.4 ± 0.8			
Waist Circumference	20–29 years	54	90.0 ± 9.6	90.0 ± 1.4	0.000	0.118	91.5 ± 1.7	Exp	0.164	0.014
(cm)	30–39 year	52	97.3 ± 11.5	97.3 ± 1.4			97.5 ± 1.4			
	40+	36	97.8 ± 8.5	97.8 ± 1.7			95.2 ± 2.5	Age	0.020	0.055
	Total	142	94.6 ± 10.7	95.0 ± 0.9			94.7 ± 0.9			
Wasit-to-Hip Ratio	20–29 years	54	0.87 ± 0.06	0.87 ± 0.01	0.000	0.151	0.89 ± 0.01	Exp	0.071	0.023
	30–39 year	52	0.92 ± 0.08	0.92 ± 0.01			0.92 ± 0.01			
	40+	36	0.94 ± 0.06	0.94 ± 0.01			0.92 ± 0.02	Age	0.081	0.036
	Total	142	0.91 ± 0.07	0.91 ± 0.01			0.91 ± 0.01			
Body Fat	40+	54	22.1 ± 5.0	22.1 ± 0.7	0.003	0.078	22.5 ± 0.9	Exp	0.396	0.005
(%)	30–39 year	52	24.3 ± 4.7	24.3 ± 0.7			24.4 ± 0.7			
	40+	36	25.6 ± 5.1	25.6 ± 0.8			24.8 ± 1.2	Age	0.226	0.021
	Total	142	23.8 ± 5.1	24.0 ± 0.4			23.9 ± 0.4			
Fat Mass	20–29 years	54	21.2 ± 7.7	21.2 ± 1.2	0.020	0.055	21.9 ± 1.5	Exp	0.479	0.004
(kg)	30–39 year	52	25.0 ± 8.3	25.0 ± 1.2			25.1 ± 1.2			
	40+	36	26.0 ± 10.3	26.0 ± 1.4			24.9 ± 2.1	Age	0.246	0.020
	Total	142	23.8 ± 8.8	24.1 ± 0.7			24.0 ± 0.8			
Lean Mass	20–29 years	54	70.0 ± 7.2	70.0 ± 1.3	0.021	0.054	70.1 ± 1.7	Exp	0.877	0.000
(kg)	30–39 year	52	73.5 ± 11.5	73.5 ± 1.3			73.5 ± 1.4			
	40+	36	67.7 ± 10.1	67.7 ± 1.6			67.5 ± 2.4	Age	0.032	0.049
	Total	142	70.7 ± 9.9	70.4 ± 0.8			70.4 ± 0.9			
Andriod Fat Distribution	20–29 years	54	24.5 ± 6.3	24.5 ± 0.9	0.000	0.122	25.3 ± 1.1	Exp	0.290	0.008
(%)	30–39 year	52	28.3 ± 6.7	28.3 ± 0.9			28.4 ± 0.9			
	40+	36	30.4 ± 6.4	30.4 ± 1.1			29.1 ± 1.6	Age	0.078	0.036
	Total	142	27.4 ± 6.9	27.7 ± 0.6			27.6 ± 0.6			
Gynoid Fat Distribution	20–29 years	54	24.0 ± 4.7	24.0 ± 0.7	0.055	0.041	23.9 ± 0.8	Exp	0.779	0.001
(%)	30–39 year	52	25.3 ± 4.4	25.3 ± 0.7			25.3 ± 0.7			
	40+	36	26.5 ± 5.5	26.5 ± 0.8			26.8 ± 1.2	Age	0.231	0.021
	Total	142	25.1 ± 4.9	25.3 ± 0.4			25.3 ± 0.4			
Visceral Adipose Tissue	20–29 years	54	77.4 ± 22.2	77.4 ± 5.1	0.000	0.226	86.1 ± 6.5	Exp	0.032	0.033
(g)	30–39 year	52	107.5 ± 46.2	107.5 ± 5.2			108.5 ± 5.2			
	40+	36	127.4 ± 41.9	127.4 ± 6.3			112.8 ± 9.1	Age	0.021	0.054
	Total	142	101.1 ± 42.4	104.1 ± 3.2			102.5 ± 3.3			

Data are expressed as means ± standard deviations (SD) with grand mean results shown as means ± standard error (SE) for the the age group effect and the effect when considering years of fire service experience as a covariate. Age = effect of age; Exp = effect of years of fire service experience; η_p_^2^ = partial eta squared effect size; n = sample size; SD = standard deviation; SE = standard error; cm = centimeters; kg = kilograms; g = grams; % = percentage. 20–29 years is the age group of those 20 to 29 years old; 30–39 years is the age group of those 30 to 39 years old; 40+ is the age group of those 40 years old or older, including those between 50–59 (7 participants) and 60+ (1 participant). Effect of Age: General linear model analysis revealed a significant overall Wilk’s Lambda for age group (*p* < 0.001, η_p_^2^ = 0.201). When considering years of fire service as a covariate, the general linear model analysis revealed no significant overall Wilk’s Lambda for age group (*p* = 0.231, η_p_^2^ = 0.079). Univariate p-levels are listed for between-subject effects with η_p_^2^ effect size values of 0.01–0.05 = small, 0.06–0.13 = medium, and >0.14 = large. Significance was determined via pairwise comparison, with LSD posthoc adjustment, and Cohen’s d effect size. ^a^ Covariates appearing in the model are evaluated at the following values: Years of fire service experience = 8.7465. Note: these results include data from one female firefighter.

**Table 3:** Blood Biomarker Results.

Variable	Age Group	*n*	Effect of Age				Effect of Age with Years of Experience Covariate^a^			
			Mean	Mean	Univariate Results		Mean	Source	Univariate Results	
			(SD)	(SE)	*p*-Value	η_p_^2^	(SE)		*p*-Value	η_p_^2^
Total Cholesterol	20–29 years	52	174.5 ± 30.4	174.5 ± 5.0	0.001	0.099	188.1 ± 6.2	Exp	0.001	0.083
(mg/dL)	30–39 year	52	191.5 ± 38.3	191.5 ± 5.0			192.9 ± 4.9			
	40+	35	204.6 ± 41.2	204.6 ± 6.1			182.3 ± 8.7	Age	0.480	0.011
	Total	139	188.4 ± 38.0	190.2 ± 3.1			187.8 ± 3.1			
High-Density Lipoprotein Cholesterol	20–29 years	52	53.3 ± 8.9	53.3 ± 1.6	0.114	0.031	53.5 ± 2.1	Exp	0.929	0.000
(mg/dL)	30–39 year	52	48.8 ± 13.9	48.8 ± 1.6			48.8 ± 1.6			
	40+	35	49.6 ± 11.9	49.6 ± 2.0			49.4 ± 2.9	Age	0.193	0.024
	Total	139	50.7 ± 11.8	50.6 ± 1.0			50.5 ± 1.0			
Low-Density Lipoprotein Cholesterol	20–29 years	52	104.6 ± 27.5	104.6 ± 4.4	0.000	0.106	116.5 ± 5.4	Exp	0.001	0.082
(mg/dL)	30–39 year	52	120.0 ± 35.4	120.0 ± 4.4			121.2 ± 4.2			
	40+	35	131.9 ± 32.0	131.9 ± 5.4			112.5 ± 7.6	Age	0.486	0.011
	Total	139	117.2 ± 33.3	118.8 ± 2.7			116.7 ± 2.7			
Apolipoprotein B	20–29 years	52	82.8 ± 18.6	82.8 ± 3.3	0.000	0.158	89.5 ± 4.2	Exp	0.010	0.048
(mg/dL)	30–39 year	52	98.0 ± 27.4	98.0 ± 3.3			98.7 ± 3.2			
	40+	35	108.4 ± 25.0	108.4 ± 4.0			97.3 ± 5.8	Age	0.207	0.023
	Total	139	94.9 ± 25.8	96.4 ± 2.1			95.2 ± 2.1			
Triglycerides	20–29 years	52	74.9 ± 37.9	74.9 ± 8.6	0.000	0.111	83.8 ± 11.0	Exp	0.197	0.012
(mg/dL)	30–39 year	52	119.8 ± 81.1	119.8 ± 8.6			120.7 ± 8.6			
	40+	35	119.1 ± 57.3	119.1 ± 10.5			104.5 ± 15.3	Age	0.019	0.057
	Total	139	102.8 ± 65.1	104.6 ± 5.3			103.0 ± 5.5			
Fasting Plasma Glucose	20–29 years	52	87.8 ± 10.1	87.8 ± 1.4	0.138	0.029	88.7 ± 1.8	Exp	0.433	0.005
(mg/dL)	30–39 year	52	90.1 ± 7.7	90.1 ± 1.4			90.2 ± 1.4			
	40+	35	92.3 ± 13.1	92.3 ± 1.7			90.8 ± 2.5	Age	0.788	0.004
	Total	139	89.8 ± 10.2	90.1 ± 0.9			89.9 ± 0.9			
Hemoglobin A1c	20–29 years	52	5.4 ± 0.2	5.4 ± 0.0	0.026	0.052	5.4 ± 0.0	Exp	0.235	0.010
(%)	30–39 year	52	5.4 ± 0.3	5.4 ± 0.0			5.4 ± 0.0			
	40+	35	5.5 ± 0.3	5.5 ± 0.0			5.5 ± 0.1	Age	0.583	0.008
	Total	139	5.4 ± 0.3	5.4 ± 0.0			5.4 ± 0.0			
FPInsulin	20–29 years	52	8.9 ± 5.4	8.9 ± 1.0	0.047	0.044	9.8 ± 1.3	Exp	0.254	0.010
(µU/mL)	30–39 year	52	11.6 ± 7.3	11.6 ± 1.0			11.7 ± 1.0			
	40+	35	12.4 ± 8.7	12.4 ± 1.2			10.9 ± 1.8	Age	0.452	0.012
	Total	139	10.8 ± 7.2	10.9 ± 0.6			10.8 ± 0.6			
HOMA-IR	20–29 years	52	1.9 ± 1.2	1.9 ± 0.3	0.024	0.053	2.2 ± 0.3	Exp	0.159	0.015
	30–39 year	52	2.6 ± 1.8	2.6 ± 0.3			2.6 ± 0.3			
	40+	35	3.0 ± 2.5	3.0 ± 0.3			2.5 ± 0.5	Age	0.549	0.009
	Total	139	2.4 ± 1.9	2.5 ± 0.2			2.5 ± 0.2			

Data are expressed as means ± standard deviations (SD) with grand mean results shown as means ± standard error (SE) for the the age group effect and the effect when considering years of fire service experience as a covariate. Age = effect of age; Exp = years of fire service experience; η_p_^2^ = partial eta squared; n = sample size; SD = standard deviation; SE = standard error; dL = deciliter; HOMA-IR = Homeostatic Model Assessment for Insulin Resistance; mg = milligrams; mL = milliliters; µU = microunits; % = percentage. 20–29 years is the age group of those 20 to 29 years old; 30–39 years is the age group of those 30 to 39 years old; 40+ is the age group of those 40 years old or older, including those between 50–59 (7 participants) and 60+ (1 participant). Effect of Age: General linear model analysis revealed a significant overall Wilk’s Lambda for age group (*p* < 0.001, η_p_^2^ = 0.148). When considering years of fire service as a covariate, the general linear model analysis revealed no significant overall Wilk’s Lambda for age group (*p* = 0.132, η_p_^2^ = 0.090). Univariate p-levels are listed for between-subject effects with η_p_^2^ effect size values of 0.01–0.05 = small, 0.06–0.13 = medium, and >0.14 = large. Significance was determined via pairwise comparison, with LSD posthoc adjustment, and Cohen’s d effect size. ^a^ Covariates appearing in the model are evaluated at the following values: Years of fire service experience = 8.6619. Note: these results include data from one female firefighter.

**Table 4:** Fitness Parameter Results.

Variable	Age Group	*n*	Effect of Age				Effect of Age with Years of Experience Covariate ^a^			
			Mean	Mean	Univariate Results		Mean	Source	Univariate Results	
			(SD)	(SE)	*p*-Value	η_p_^2^	(SE)		*p*-Value	η_p_^2^
CPXT Time-to-Exhaustion	20–29 years	54	12.1 ± 1.5	12.1 ± 0.2	0.000	0.206	11.6 ± 0.3	Exp	0.008	0.051
(minutes)	30–39 year	51	10.8 ± 1.5	10.8 ± 0.2			10.8 ± 0.2			
	40+	34	10.2 ± 1.5	10.2 ± 0.3			10.9 ± 0.4	Age	0.034	0.049
	Total	139	11.1 ± 1.7	11.0 ± 0.1			11.1 ± 0.1			
Maximal Oxygen Uptake	20–29 years	54	42.7 ± 6.2	42.7 ± 0.8	0.000	0.211	41.1 ± 1.1	Exp	0.015	0.043
(ml/kg/min)	30–39 year	51	37.4 ± 6.3	37.4 ± 0.9			37.2 ± 0.9			
	40+	34	35.1 ± 6.0	35.1 ± 1.1			37.8 ± 1.5	Age	0.016	0.060
	Total	139	38.9 ± 6.9	38.4 ± 0.5			38.7 ± 0.5			
Sit-Ups	20–29 years	54	44.5 ± 8.0	44.5 ± 1.3	0.085	0.036	41.3 ± 1.6	Exp	0.002	0.070
(repetitions)	30–39 year	51	40.5 ± 11.1	40.5 ± 1.3			40.2 ± 1.3			
	40+	34	41.1 ± 9.7	41.1 ± 1.6			46.6 ± 2.3	Age	0.062	0.040
	Total	139	42.2 ± 9.7	42.0 ± 0.8			42.7 ± 0.8			
Push-Ups	20–29 years	54	60.1 ± 15.1	60.1 ± 2.3	0.000	0.160	57.0 ± 2.9	Exp	0.089	0.021
(repetitions)	30–39 year	51	49.1 ± 17.4	49.1 ± 2.4			48.8 ± 2.4			
	40+	34	41.7 ± 19.6	41.7 ± 2.9			47.0 ± 4.3	Age	0.082	0.036
	Total	139	51.6 ± 18.5	50.3 ± 1.5			51.0 ± 1.5			
Sit-and-Reach	20–29 years	54	17.1 ± 3.3	17.1 ± 0.4	0.000	0.117	16.7 ± 0.6	Exp	0.179	0.013
(inches)	30–39 year	51	15.4 ± 3.1	15.4 ± 0.5			15.3 ± 0.5			
	40+	34	14.2 ± 3.2	14.2 ± 0.6			15.0 ± 0.8	Age	0.158	0.027
	Total	139	15.8 ± 3.4	15.6 ± 0.3			15.7 ± 0.3			
Handgrip Strength	20–29 years	54	57.6 ± 6.5	57.6 ± 1.0	0.116	0.031	57.4 ± 1.2	Exp	0.858	0.000
(kg)	30–39 year	51	60.4 ± 7.9	60.4 ± 1.0			60.4 ± 1.0			
	40+	34	58.1 ± 7.1	58.1 ± 1.2			58.3 ± 1.8	Age	0.116	0.031
	Total	139	58.7 ± 7.3	58.7 ± 0.6			58.7 ± 0.6			

Data are expressed as means ± standard deviations (SD) with grand mean results shown as means ± standard error (SE) for the the age group effect and the effect when considering years of fire service experience as a covariate. Age = effect of age; Exp = years of fire service experience; η_p_^2^ = partial eta squared; n = sample size; SD = standard deviation; SE = standard error; CPXT = cardiopulmonary exercise test; kg = kilogram; ml = milliliter; min = minute. 20–29 years is the age group of those 20 to 29 years old; 30–39 years is the age group of those 30 to 39 years old; 40+ is the age group of those 40 years old or older, including those between 50–59 (7 participants) and 60+ (1 participant). Effect of Age: General linear model analysis revealed a significant overall Wilk’s Lambda for age group (*p* < 0.001, η_p_^2^ = 0.180). When considering years of fire service as a covariate, the general linear model analysis revealed a significant overall Wilk’s Lambda for age group (*p* = 0.008, η_p_^2^ = 0.096). Univariate p-levels are listed for between-subject effects with η_p_^2^ effect size values of 0.01–0.05 = small, 0.06–0.13 = medium, and >0.14 = large. Significance was determined via pairwise comparison, with LSD posthoc adjustment, and Cohen’s d effect size. ^a^ Covariates appearing in the model are evaluated at the following values: Years of fire service experience = 8.6115. Note: these results include data from one female firefighter.

## Data Availability

Data is contained within the article.

## Abbreviations

The following abbreviations are used in this manuscript:

ACSM	American College of Sports Medicine
ApoB	Apolipoprotein B
BSL-2	Biosafety Level 2
BMI	Body Mass Index
CVD	Cardiovascular Disease
DEXA	Dual-Energy X-Ray Absorptiometry Scan
EDTA	Ethylenediaminetetraacetic Acid
GLM	General Linear Model
HbA1c	Hemoglobin-A1c
HDL-C	High-Density Lipoprotein
HOMA-IR	Homeostatic Model Assessment for Insulin Resistance
LDL-C	Low-Density Lipoprotein Cholesterol
CPXT	Maximal Cardiopulmonary Exercise Test
η_p_^2^	Partial Eta Squared
SST	Serum Separation Tubes
SD	Standard Deviations
SE	Standard Error
TTE	Time-To-Exhaustion
TC	Total Cholesterol
TAG	Triglycerides
VO_2_max	Maximal Oxygen Uptake
